# Xolremdi (Mavorixafor): a breakthrough in WHIM syndrome treatment – unraveling efficacy and safety in a rare disease frontier

**DOI:** 10.1097/MS9.0000000000002590

**Published:** 2024-09-24

**Authors:** Fatima Nadeem, Laiba Shakeel, Aymar Akilimali

**Affiliations:** a Department of Internal Medicine, Dow University of Health Sciences, Karachi, Pakistan; b Department of Research, Medical Research Circle, Goma, Democratic Republic of the Congo

## Introduction

WHIM syndrome is a rare primary immunodeficiency (PI) and chronic neutropenic disorder characterized by the dysfunction of the immune system and difficulties in combating infections. It is caused by dysfunction of the CXCR4 receptor due to mutations in the CXCR4 gene. The acronym ‘WHIM’ represents the syndrome’s four classic features: susceptibility to warts induced by human papillomavirus infection, hypogammaglobulinemia, infections, and myelokathexsis^1^​. The exact prevalence of WHIM syndrome is unknown, but it is estimated to occur in approximately one in every five million live births. Since myelokathexis was first described in 1964, and until the molecular basis of WHIM syndrome was discovered in 2003, over 100 cases have been documented in the medical literature^2^. One study reported that the syndrome is more prevalent in female patients, with most cases reported from North America and Eastern Europe. To date, only one fatality has been recorded, attributed to complications from hematopoietic stem cell transplantation^2^.

This article provides a comprehensive overview of WHIM syndrome, including its pathophysiology, clinical manifestations, and current treatment options, and sets the stage for discussing the novel treatment, mavorixafor (Xolremdi), a recently approved FDA drug, once-daily oral therapy for treating WHIM syndrome. It also provides a comprehensive understanding of its action and compelling potential in effectively managing the condition.

## Pathophysiology

WHIM syndrome is often caused by autosomal dominant pathogenic variants in the CXCR4 gene located on chromosome 2q22. This gene encodes a G-protein-coupled chemokine receptor that is highly expressed in embryonic stem and progenitor cells. It regulates the development of the hematopoietic, cardiovascular, nervous, and reproductive systems, highlighting its essential role in various biological functions and its potential pathological significance^3^. In cases with CXCR4 gain-of-function (GOF) variants, CXCR4 internalization decreases, prolonging its interaction with its sole chemokine ligand, CXCL12. This results in hyperactive signaling and impairs myeloid and lymphoid cell responses to CXCL12 gradients, hindering their migration from the bone marrow to the periphery or sites of inflammation. Consequently, neutrophils over-mature in the bone marrow, leading to ‘myelokathexis’, which is characterized by the retention of these cells in the marrow^2^. This bone marrow pathology affects neutrophils and disrupts the development and migration of B and T cells. Additionally, the overexpression of the CXCR4 gene promotes various aspects of tumor progression, including growth, invasion, angiogenesis, metastasis, relapse, and resistance to therapy^2^.

### Clinical manifestations

WHIM syndrome has traditionally been diagnosed using clinicopathological criteria, with a bone marrow biopsy revealing the characteristic feature known as myelokathexis^4^. Neutropenia, a key laboratory feature of WHIM syndrome, typically appears early in life, often at birth. However, it can occasionally resolve during infections, which may lead to a delayed diagnosis^5,6^. Although warts develop in 61% of cases, they may be absent in the early stages of life, which can complicate the diagnosis of WHIM syndrome^7^. Genetic testing, especially for gain-of-function variants in the C-terminal region of the CXCR4 gene, is increasingly used to confirm WHIM syndrome clinically. This approach provides a noninvasive and accessible alternative to bone marrow biopsy for confirming myelokathexis and may facilitate earlier diagnosis^2^. Individuals with WHIM syndrome experience extremely low neutrophil (neutropenia) and lymphocyte (lymphopenia) counts, making them highly vulnerable to severe bacterial and viral infections^1^.

In a study assessing WHIM syndrome progression in 66 patients, most disease manifestations appeared within the first year of clinical onset. The majority of patients exhibited laboratory abnormalities, including noncyclic neutropenia, lymphopenia, and hypogammaglobulinemia. Infections were prevalent, with otitis media and pneumonia being particularly common. The combined lifetime prevalence of bacterial meningitis and bacteremia leading to sepsis was 13%. Additionally, 20% of patients experienced infection-related organ damage, such as bronchiectasis and bronchiolectasis from recurrent pneumonia and hearing loss from recurrent otitis^2^.

HPV-related symptoms were observed in 42% of patients, with 40% having warts on various parts of their bodies. Although no HPV-related cancers were documented, two patients had malignancies unrelated to HPV: an infant with a melanotic neuroectodermal tumor and an adult with basal cell carcinoma. Autoimmune complications were present in 21% of patients. Heart abnormalities, including aortic valve insufficiency, tricuspid valve insufficiency, right-sided aortic arch, tetralogy of Fallot, and Wolff-Parkinson-White (WPW) syndrome, were found in 17% of patients. Additionally, 3% of patients had minor malformations, such as maxillofacial anomalies^2^. These findings underscore the multifaceted nature of WHIM syndrome.

### Current treatment

#### Supportive therapies

Current therapeutic options for WHIM syndrome are primarily supportive and include granulocyte colony-stimulating factor (G-CSF), immunoglobulin replacement therapy (IgGRT), and antimicrobial prophylaxis.

In a trial involving 54 individuals, 30 were treated with G-CSF. About half of these patients experienced improvements, such as increased absolute neutrophil counts (ANC) and fewer infection-related issues. However, 22% of patients had improved ANC counts but did not see a reduction in infection susceptibility. For 26% of patients, G-CSF neither improved ANC counts nor reduced infection frequency.

Of the 54 patients, 29 received IgGRT, which effectively reduced infection rates in 74% of cases. Partial responses were noted in 22%, while 4.35% showed no response. Additionally, 23 patients received antibiotic prophylaxis, with 80% experiencing reduced infection rates, 10% showing partial responses, and another 10% showing no response^2,3^.

#### Treatment of warts

Primary treatments for warts typically include topical salicylic acid and retinoids, which are often ineffective in most cases. Consequently, alternative approaches may be required, such as surgical interventions or other medical therapies, including imiquimod, cryotherapy, laser treatment, or diathermocoagulation^3^.

#### HPV vaccination

Although there is no vaccine specifically for WHIM syndrome, prophylactic administration of the HPV vaccine to young WHIM patients has shown promise in providing protective immunity, allowing them to remain wart-free for several years. However, the long-term effectiveness of the vaccine is limited in WHIM patients due to impaired memory B cell development or maintenance, leading to an early decline in antibody response to vaccine antigens^3^. As a result, individuals with WHIM syndrome have still contracted influenza despite being vaccinated against it^8^.

#### Selective CXCR4 antagonist

One potential drug for WHIM syndrome is Plerixafor, a selective CXCR4 antagonist approved by the FDA for mobilizing hematopoietic stem cells from bone marrow to blood for transplantation in cancer patients. Plerixafor has emerged as a promising targeted therapy for WHIM syndrome due to its ability to modulate excessive CXCR4 signaling. Initial clinical trials have shown its safety and efficacy in improving infections, warts, and neutropenia, though it does not fully restore immunoglobulin levels. However, in a phase 3 crossover trial, Plerixafor was found to be nonsuperior to G-CSF in reducing the total infection severity score. It was also inferior in maintaining an absolute neutrophil count (ANC) above 500 cells/μl, but it was superior in maintaining an absolute lymphocyte count (ALC) above 1000 cells/μl^9,10^.

#### Hematopoietic stem cell transplantation (HSCT)

Seven patients underwent hematopoietic stem cell transplantation (HSCT) as a definitive treatment for WHIM syndrome, with successful engraftment in all but one case. In that instance, the patient tragically died due to infectious complications following graft rejection after the third HSCT attempt. At a median follow-up of 6.7 years, all six surviving patients maintained full donor chimerism^11^. While HSCT shows potential as a treatment, its long-term efficacy remains uncertain. It has been considered early in the disease’s progression to prevent complications such as disfiguring warts, chronic lung disease, and increased susceptibility to malignancy later in life^3^.

#### Gene editing

Lastly, gene editing may also offer a viable cure for WHIM syndrome, as demonstrated by the clinical cure observed in one patient following a chromothriptic deletion of the disease allele in hematopoietic stem cells^12^.

### Mavorixafor as the drug therapy

**Figure 1:**
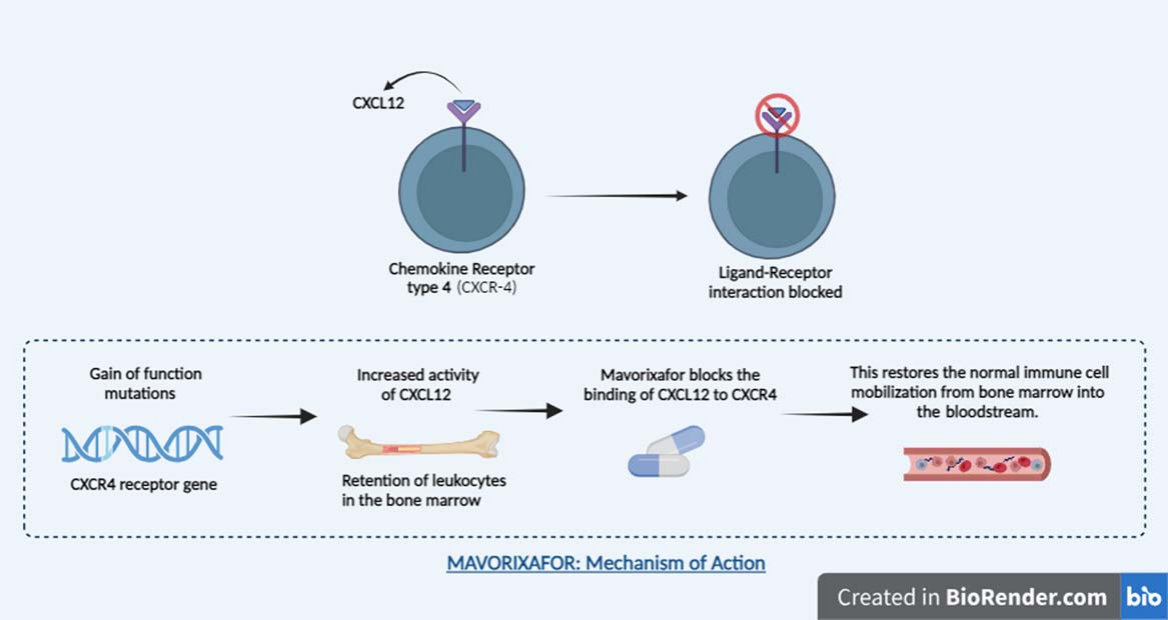
Mechanism of action of mavorixafor.

Mavorixafor, an orally administered medication approved for patients aged 12 and older with WHIM syndrome, has significantly transformed the treatment landscape. As a potent CXCR4 receptor antagonist, mavorixafor plays a crucial role in regulating the trafficking of leukocytes between the bone marrow and peripheral circulation. The ligand stromal-derived factor-1α (SDF-1α), also known as CXC Chemokine Ligand 12 (CXCL12), binds to CXCR4, guiding the movement of immune cells. In patients with WHIM syndrome, gain-of-function mutations in the CXCR4 receptor gene lead to heightened responsiveness to CXCL12, causing leukocytes to be retained in the bone marrow. Mavorixafor disrupts this pathological process by blocking the binding of CXCL12 to CXCR4, thereby reducing the exaggerated response of the mutated receptor and normalizing immune cell mobilization. This action promotes the release of neutrophils and lymphocytes from the bone marrow into the bloodstream (Fig. 1). Notably, mavorixafor’s mechanism of action is effective not only against mutated CXCR4 variants associated with WHIM syndrome but also extends to the wild-type receptor^13^.

### Clinical trials

The X4P-001-MKKA study reported the safety and preliminary efficacy of mavorixafor in a phase 2 extension trial involving eight adult patients with genetically confirmed WHIM syndrome, observed for up to 28.6 months. The study included patients aged 18 years and older who had a pathogenic CXCR4 mutation and an absolute neutrophil count (ANC) ≤400/μl and/or an absolute lymphocyte count (ALC) ≤650/μl. Mavorixafor was well tolerated, with no serious adverse events related to the treatment.

After a median follow-up of 16.5 months, dose-dependent increases in both ANC and ALC were observed. For doses of 300 mg/day or higher, the ANC remained above 500 cells/μl for a median of 12.6 h, and the ALC stayed above 1000 cells/μl for up to 16.9 h. The annual infection rate for patients treated with 400 mg once daily decreased to 2.14 events, compared to 4.63 events in the prior 12 months. Additionally, there was an average reduction of 75% in the number of cutaneous warts^14^.

Similarly, in a randomized (1:1), double-blind, placebo-controlled, phase 3 trial, 31 participants aged ≥12 years with WHIM syndrome and an absolute neutrophil count (ANC) ≤400/μl were enrolled. They were given either mavorixafor (*n*=14) 400 mg once daily or placebo (*n*=17) for 52 weeks^10^. Primary and secondary endpoints were established in the study. The primary endpoint was the time (hours) above the ANC threshold of ≥500/μl (TATANC) over 24 h, evaluated every 3 months for 52 weeks. One of the secondary endpoints was the TAT absolute lymphocyte count ≥1000/μl (TATALC). The least squares (LS) mean TATANC for mavorixafor was 15.0 h, compared to 2.8 h for the placebo. The LS mean TATALC was 15.8 h for mavorixafor, versus 4.6 h for the placebo.

After a median follow-up of 359 days for the mavorixafor group and 364 days for the placebo group, mavorixafor significantly increased the mean ANC and ALC above the 500 and 1000 cell/μl thresholds, respectively, with a 2.8-fold to 3.3-fold increase in absolute monocyte count, white blood cell (WBC) counts, ANC, and ALC after 364 days. No adverse events associated with the treatment led to discontinuations or fatalities.

The trial demonstrated that mavorixafor was generally well-tolerated and effectively reduced the frequency, severity, and duration of infections, as well as the need for antibiotics during infections. Significant reductions in total infection score and annualized infection rate were observed within 3 months of treatment, with further improvements noted between 6 and 12 months. No grade 3 or higher infections occurred during the study. Some patients on mavorixafor experienced side effects such as vomiting, dyspepsia, and nausea, each reported by one participant^10^ (summarised in Table 1).

**Table 1:** Key discoveries from mavorixafor’s trial.

Study ID	Drug	Phase	Sample size	Outcomes	Adverse effect
NCT03005327	Patients received escalated doses of mavorixafor based on mean AUCANC and AUCALC: 50 mg (*n*=2), 100 mg (*n*=4), 150 mg (*n*=2), 200 mg (*n*=3), 300 mg (*n*=7), and 400 mg (*n*=3). Not all patients received all doses^14^	Phase 2	We enrolled eight patients (mean age at inclusion was 33 years) for up to a maximum duration of 28.6 months five patients followed with an extension study conducted in two clinical trial sites located in Australia and the United States^14^	The primary endpoint, used to determine dose escalation, was the mean value of the threshold-adjusted AUCANC and/or AUCALC collected over 24 h. A dose-dependent increase in the mean WBC, ANC, ALC, AMC, AUCANC, and AUCALC was observed in the mavorixafor group^14^	Seven patients (87.5%) experienced at least one treatment-emergent adverse event (TEAE), while three patients experienced 11 grade 1 related TEAEs, including nausea, nasal dryness, dry mouth, dyspepsia, conjunctivitis, and dermatitis psoriasiform rash^14^
NCT03995108	Thirty-one patients were were randomly allocated and were given either mavorixafor (*n*=14) 400 mg once daily or placebo (*n*=17) for 12 months (52 weeks)^10^	Phase 3	Thirty-one participants aged ≥12 years with WHIM syndrome and an absolute neutrophil count (ANC) ≤400/μl were enrolled^10^	Time (hours) above ANC threshold ≥500/μl (TATANC; over 24 h) was the primary endpoint which was evaluated every 3 months for 52 weeks. The TAT absolute lymphocyte count ≥1000/μl (TATALC) was one of the secondary endpoints. Mavorixafor for least squares (LS) mean TATANC was 15.0 h, placebo 2.8 h, and LS mean TATALC was 15.8 h, placebo 4.6 hours^10^	No adverse events associated with treatment led to discontinuations or fatalities. Some Mavorixafor patients experienced side effects like vomiting, dyspepsia, and nausea, each occurring in one participant^10^

## Recommended dosage and adverse effects

Xolremdi, with the active ingredient mavorixafor, is recommended at a dosage of 400 mg for individuals weighing more than 50 kg and 300 mg for those weighing 50 kg or less, taken orally once daily^13^.

Xolremdi can prolong the QTc interval in a concentration-dependent manner, a risk that increases when used alongside other medications that either elevate Xolremdi levels or are known to prolong the QT interval. To mitigate this risk, it is important to address modifiable factors like hypokalemia, assess baseline QTc, and monitor QTc during treatment, particularly in patients who have additional risk factors for QTc prolongation. This monitoring helps prevent serious conditions like torsade de pointes, other significant arrhythmias, and sudden death.

Due to its mechanism of action, Xolremdi is expected to cause harm to a fetus if administered during pregnancy, though there is no available data on its use in pregnant women to confirm this risk. Additionally, Xolremdi is not recommended for patients with moderate to severe hepatic or renal impairment^13^.

### Limitations

While Xolremdi (mavorixafor) has shown promising results in the treatment of WHIM syndrome, there are several limitations to its current understanding and application. Clinical trials for Xolremdi have primarily involved small patient cohorts, which limits the generalizability of the findings to larger populations. To validate these results on a broader scale, larger and multicenter trials among diverse populations are necessary.

The side effects of Xolremdi, such as vomiting, dyspepsia, and nausea, while not severe enough to cause discontinuations, could affect patient compliance and overall quality of life. Additionally, there is a lack of long-term efficacy and safety data for Xolremdi beyond the scope of the studied protocols. Therefore, extensive longitudinal studies are needed to identify any potential long-term adverse effects.

As a novel treatment for a rare condition, the widespread adoption and availability of Xolremdi could be hampered by challenges related to accessibility and affordability on a global scale. Efforts to enhance production and reduce costs will be crucial in ensuring broader access to this treatment.

Xolremdi effectively addresses neutropenia and lymphopenia in WHIM syndrome, but it may not fully alleviate all other symptoms associated with the condition, such as chronic infections and HPV-related malignancies. Additionally, there is a potential risk of patients developing resistance to Xolremdi over time, as is common with many targeted therapies.

Moreover, the associated risks for specific patient populations, particularly pregnant women, and those with renal or hepatic impairments, highlight the need for careful monitoring by healthcare professionals. Continuous vigilance and, possibly, combination therapies may be required to maintain the drug’s efficacy and manage any emerging challenges.

## Conclusion

In conclusion, the FDA approval of Xolremdi marks a significant milestone in medical innovation. It is a promising and generally well-tolerated drug, with no reported discontinuations due to its safety profile. By targeting the dysfunction in the CXCR4 pathway, Xolremdi effectively increases the levels of mature neutrophils and lymphocytes in circulation, addressing the root cause of WHIM syndrome. The clinical findings highlight its efficacy and safety, solidifying its potential as a valuable therapeutic option for this rare condition.

As we look ahead, ongoing research and clinical experience will be crucial in further understanding its long-term benefits and refining its use in practice. The approval of Xolremdi offers renewed hope for patients and clinicians, opening up new possibilities for improved outcomes and a better quality of life. To maximize its impact, efforts should be made to increase production, ensure global accessibility, and make it cost-effective. This would not only enhance the lives of those affected by WHIM syndrome but also reduce the burden on healthcare systems. Xolremdi’s approval is a promising step forward in the treatment of WHIM syndrome, paving the way for a brighter future for patients worldwide.

## Ethical approval

No patient was involved in this type of study, therefore no ethical approval was required.

## Consent

The study is an editorial and was not done on patients or volunteers, therefore no written consent was required.

## Source of funding

None.

## Author contribution

L.S.: conceptualization and project administration; F.N.: original draft of manuscript; A.A.: reviewing and editing the manuscript; L.S.: visualization.

## Conflicts of interest disclosure

The authors declare that they have no financial conflict of interest with regard to the content of this report.

## Research registration unique identifying number (UIN)

1. Name of the registry: not applicable.
2. Unique identifying number or registration ID: not applicable.
3. Hyperlink to your specific registration (must be publicly accessible and will be checked): not applicable.

## Guarantor

Aymar Akilimali.

## Data availability statement

Not available.

## Provenance and peer review

Not commissioned, externally peer-reviewed.
